# High-Grade Atrioventricular Block Reveals Rare Transthyretin Cardiac Amyloidosis With Unique Hemodynamics

**DOI:** 10.7759/cureus.58166

**Published:** 2024-04-13

**Authors:** Zeyad J Rifai, Mohamad H Sukkari, Samie Gilani, Abhishek Kulkarni

**Affiliations:** 1 Department of Internal Medicine, Southern Illinois University School of Medicine, Springfield, USA; 2 Department of Cardiology, Southern Illinois University School of Medicine, Springfield, USA

**Keywords:** tafamadis, severe hypertension, pyp scintigraphy, neurogenic orthostatic hypotension, attr cardiac amyloidosis, permanent pacemaker implantation (ppm), high-degree av block

## Abstract

Atrioventricular (AV) block is a common cardiac conduction disorder that is frequently encountered in clinical practice; however, the association with rare systemic conditions such as transthyretin amyloidosis (ATTR) is heavily underdiagnosed. ATTR amyloidosis is a systemic disorder characterized by the deposition of abnormal transthyretin protein fibrosis in various organs including the heart and vasculature, resulting in progressive organ dysfunction. We present a rare case of high-grade AV block unveiling ATTR cardiac amyloidosis with unusual hemodynamics, specifically severe supine hypertension with severe orthostatic hypotension. These findings posed a diagnostic challenge, underscoring the importance of a comprehensive diagnostic approach and meticulous review of medical history. Following pacemaker placement and the diagnosis of ATTR cardiac amyloidosis, our patient was started on a Tafamidis regimen.

## Introduction

Cardiac amyloidosis results in structural and functional impairment of the heart and is characterized by the infiltration of the myocardium with abnormal protein fibrils [1]. Dozens of amyloidogenic proteins have been identified, but the most commonly encountered forms in clinical practice are systemic immunoglobulin light chain and transthyretin amyloidosis (ATTR). ATTR cardiac amyloidosis is further classified into hereditary or variant, and senile or wild-type forms [1]. Despite their etiological differences, both forms of ATTR cardiac amyloidosis manifest as progressive infiltrative cardiomyopathy, characterized classically by myocardial stiffening, diastolic dysfunction, and subsequent heart failure [2,3]. However, the clinical spectrum of ATTR cardiac amyloidosis ranges from subclinical disease with incidental findings on imaging studies to overt heart failure with life-threatening complications [4]. The true prevalence of ATTR cardiac amyloidosis is unknown. Due to the insidious onset and nonspecific clinical manifestations, ATTR cardiac amyloidosis often eludes early diagnosis, resulting in delayed intervention and poor prognosis.

High-grade atrioventricular (AV) block is characterized by impairment in the conduction system between the atria and ventricles of the heart. This critical class of arrhythmias has increased in prevalence in our aging population, necessitating prompt recognition given the potential precipitation of adverse outcomes including syncope, heart failure, and even sudden cardiac death [4]. We describe an unusual case of high-grade AV block with unique hemodynamics secondary to ATTR cardiac amyloidosis.

## Case presentation

**Figure 1:**
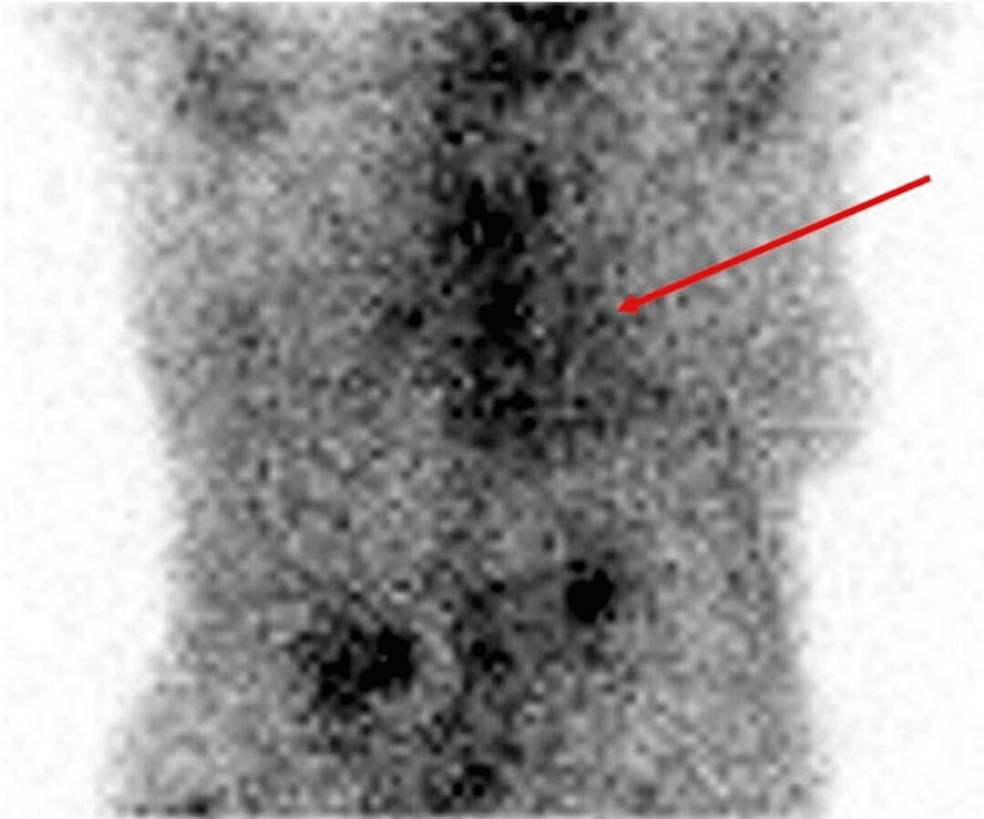
Cardiac uptake of 99mTc-PYP was evaluated using a semiqualitative visual scoring method in relation to bone uptake at 1 hour and at 3 hours after injection: positive for grade II ATTR cardiac amyloid Red arrow pointing to the site of myocardial uptake, positive for ATTR cardiac amyloid. ATTR, transthyretin amyloidosis; 99mTc-PYP, technetium-99m pyrophosphate

**Figure 2:**
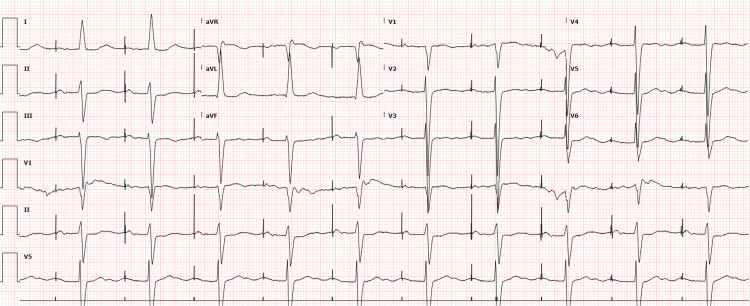
Pseudoinfarct pattern without low-voltage criteria, and left ventricular hypertrophy

A 93-year-old female presented with fatigue, weakness, and headache and was diagnosed with high-grade AV block, resulting in pacemaker placement. Her clinical presentation evolved into a more unusual cardiovascular condition. Following pacemaker placement, the patient experienced supine hypertension with significant orthostatic hypotension, with a difference of eighty systolic blood pressure points on average. After aggressive volume resuscitation, the patient continued to display profound orthostatic hypotension with significant supine hypertension, prompting an in-depth evaluation. There was no evidence of adrenal insufficiency, infection, or volume depletion, and all vasoactive medications were discontinued. The patient's brain natriuretic peptide was elevated, and a transthoracic echocardiogram was obtained, which revealed left ventricular ejection fraction of 63% with evidence of left ventricular thickening, elevated left ventricular end diastolic pressure of 14 mmHg, and a grade II diastolic dysfunction with an early diastolic mitral inflow velocity to early diastolic mitral annulus velocity (E/e’ ratio) of 16. There was no evidence of systolic anterior motion of the mitral valve or other signs of left ventricular outflow tract obstruction, as evidenced by the measured left ventricular outflow tract peak gradient, which was 4 mmHg (Video 1). The aortic valve area was 2.37 cm2, aortic valve peak velocity was 1.34 meter/second, aortic valve peak gradient was 7.2 mmHg, and aortic valve mean gradient was 3.5 mmHg; these findings suggest no significant aortic valve sclerosis or stenosis. There were no notable wall motion segment abnormalities. Methodical review of the patient's medical history revealed distant bilateral carpal tunnel release surgery, with surgery dates separated by more than 10 years. This raised our clinical suspicion for cardiac amyloidosis, prompting pursuit of a technetium-99m pyrophosphate (99mTc-PYP) cardiac scintigraphy, revealing grade II myocardial 99mTc-PYP uptake, with positive SPECT imaging performed three hours after uptake ultimately confirming cardiac involvement of transthyretin (ATTR) amyloidosis (Figure 1). Of note, the patient's electrocardiogram revealed evidence of left ventricular hypertrophy per voltage criteria and “pseudoinfarct” pattern without typified low voltage QRS (Figure 2). There was no evidence of monoclonal protein on serum or urine immunofixation. The patient was started on Tafamidis, discharged on a stable blood pressure regimen, and scheduled for close follow-up with cardiology.

**Video 1:** Parasternal long-axis view of the heart, without evidence of systolic anterior motion of the mitral valve Left ventricular outflow tract obstructions may result in orthostatic hypotensive symptoms. However, the left ventricle peak gradient calculated from this transthoracic echocardiogram did not reveal evidence of left ventricular outflow tract obstruction.

## Discussion

Wild-type ATTR cardiac amyloidosis involves the deposition of misfolded transthyretin protein in the myocardium and arterial walls, disrupting normal cardiac and vascular architecture and resulting in impaired cardiac function [1]. The reduced vascular compliance likely contributed to the increased systolic blood pressure in the supine position in our patient. ATTR can also deposit amyloid fibrils within the autonomic nervous system, raising our suspicion of amyloid infiltration in the carotid and aortic baroreceptors, ultimately leading to profound orthostatic hypotension. This leads to a discussion regarding the role of Tafamidis. Maurer et al.’s study focused on Tafamidis outcomes in patients with cardiac amyloidosis and noted a 13.4% absolute reduction in overall mortality and a 22% absolute reduction in yearly cardiovascular hospitalizations at 30 months when compared to placebo. Interestingly, the study highlighted that the overall benefit of Tafamidis was more pronounced after 18 months in relation to survival and cardiovascular hospitalizations [5]. This raises the question of Tafamidis’ role in cardiac remodeling, resulting in reduced decline in functional capacity. The René et al study was consistent with Maurer et al.’s study, suggesting left ventricular ejection fraction benefit on cardiac magnetic resonance following the initiation of Tafamidis [6]. Of note, previous placement of a cardiac pacemaker was part of the exclusion criteria for Maurer et al.’s study; however, the mortality benefit and reduction in functional capacity seen with Tafamidis ultimately guided our management.

Patients with ATTR cardiac amyloidosis can present with varying degrees of heart block given the extracellular deposition of amyloid fibrils within the myocardium, leading to disarrangement of the myocardial architecture [4,7]. Diastolic dysfunction results in increased left ventricular end-diastolic pressure and left atrial pressures, and this increase in left atrial pressure results in left atrial dilatation increasing the likelihood of developing atrial arrhythmias [1,7]. There is also a neurotoxic effect of transthyretin that is believed to have a concentration-dependent effect [6]. The mechanism of transthyretin neurotoxicity is postulated to be multifactorial, involving apoptosis as well as intracellular calcium signaling disruption [6]. This explains the ability of ATTR to disrupt the cardiac conduction system given the conduction system interplay between myocardial and neuronal tissue [8,9]. A study conducted by Donnellan et al. focusing on examining the incidence and prevalence of high-grade AV block requiring pacemaker placement noted a 9.5% incidence of high-grade AV block requiring pacemaker implantation secondary to ATTR cardiac amyloidosis. The majority of the ATTR cardiac amyloidosis findings occurred after placement of a pacemaker [4]. These data strongly indicate that high-grade AV block may frequently result from ATTR cardiac amyloidosis and is significantly underdiagnosed in clinical practice.

While endomyocardial biopsies remain the gold standard method for diagnosing cardiac amyloidosis and distinguishing between its various subtypes, the use is impeded due to procedural constraints. As an alternative, radionuclide cardiac imaging has gained prominence as a preferred diagnostic tool for identifying ATTR, increasingly supplanting traditional biopsy approaches. The 99mTc-PYP scintigraphy has a sensitivity of 99% and specificity of 86% in the absence of evidence of monoclonal gammopathy value for ATTR cardiac amyloidosis [10]. However, single-photon emission computed tomography (SPECT) is essential in the diagnostic process given the possibility of false-positive results seen in 99mTc-PYP scintigraphy [10]. In Poterucha et al.’s study, in 25 patients with grade II 99mTc-PYP uptake scans, eight (32%) cases had negative SPECTs, eight (32%) cases had blood pooling, and nine (36%) cases had true myocardial uptake [10]. Genetic testing is an encouraging trend toward individualized medicine and has a significant role in the diagnostic process of hereditary amyloidosis. Given her age, our patient and her family deferred genetic testing as it is assumed that the patient has a senile or wild-type variant of ATTR cardiac amyloidosis. Consequently, when encountering a patient with high-grade AV block, clinicians should consider ATTR cardiac amyloidosis as a potential etiology warranting its inclusion as a prominent differential diagnosis. This recognition is crucial for timely diagnosis and appropriate management, emphasizing the need for heightened awareness among healthcare professionals regarding the association between high-grade AV block and ATTR cardiac amyloidosis. This discussion is limited as this is a single case of rare hemodynamic findings secondary to ATTR cardiac amyloidosis following pacemaker placement.

## Conclusions

Our case was complicated by unique hemodynamics requiring an in-depth evaluation of the patient's medical history, uncovering the true etiology of the patient's underlying disease. This case highlights the significance of considering ATTR cardiac amyloidosis in patients with high-grade AV block, as this potential underlying etiology is significantly underdiagnosed. Radionuclide cardiac imaging, such as 99mTc-PYP scintigraphy, is a valuable non-invasive diagnostic tool with high sensitivity and specificity for identifying ATTR cardiac amyloidosis. Prompt recognition of this association is crucial for timely diagnosis and appropriate management, underscoring the importance of heightened clinical suspicion and comprehensive evaluation in patients with high-grade AV block.
